# Micropollutants and the Restructuring of Microbial Resilience in the Anthropocene

**DOI:** 10.1111/gcb.71073

**Published:** 2026-08-24

**Authors:** Despo Fatta‐Kassinos, Andrea Naziri, Celia Manaia, Luis Pedro Coelho

**Affiliations:** ^1^ Department of Civil and Environmental Engineering and Nireas‐International Water Research Center, School of Engineering University of Cyprus Nicosia Cyprus; ^2^ Universidade Católica Portuguesa CBQF—Centro de Biotecnologia e Química Fina, Laboratório Associado, Escola Superior de Biotecnologia Porto Portugal; ^3^ Centre for Microbiome Research, School of Biomedical Sciences Queensland University of Technology (QUT), Translational Research Institute Woolloongabba Queensland Australia

**Keywords:** anthropogenic chemical mixtures, ecological memory, environmental microbiomes, microbial adaptation, microbial resilience, micropollutants, resilience displacement, wastewater–environment continuum

## Abstract

Conceptual framework linking anthropogenic chemical complexity to the restructuring of microbial resilience in the Anthropocene. Persistent, low‐dose chemical mixtures can reorganize microbial coordination networks, regulatory and metabolic processes, and ecological memory, thereby altering functional redundancy, adaptive capacity, and disturbance–recovery dynamics. The lower panel illustrates a possible threshold‐like transition where reinforcing feedbacks support alternative functional regimes, rather than a universal response to chronic chemical pressure.

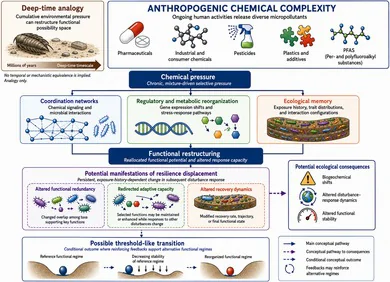

## 1 Deep‐Time Analogy and Chemical Complexity

**FIGURE 1:**
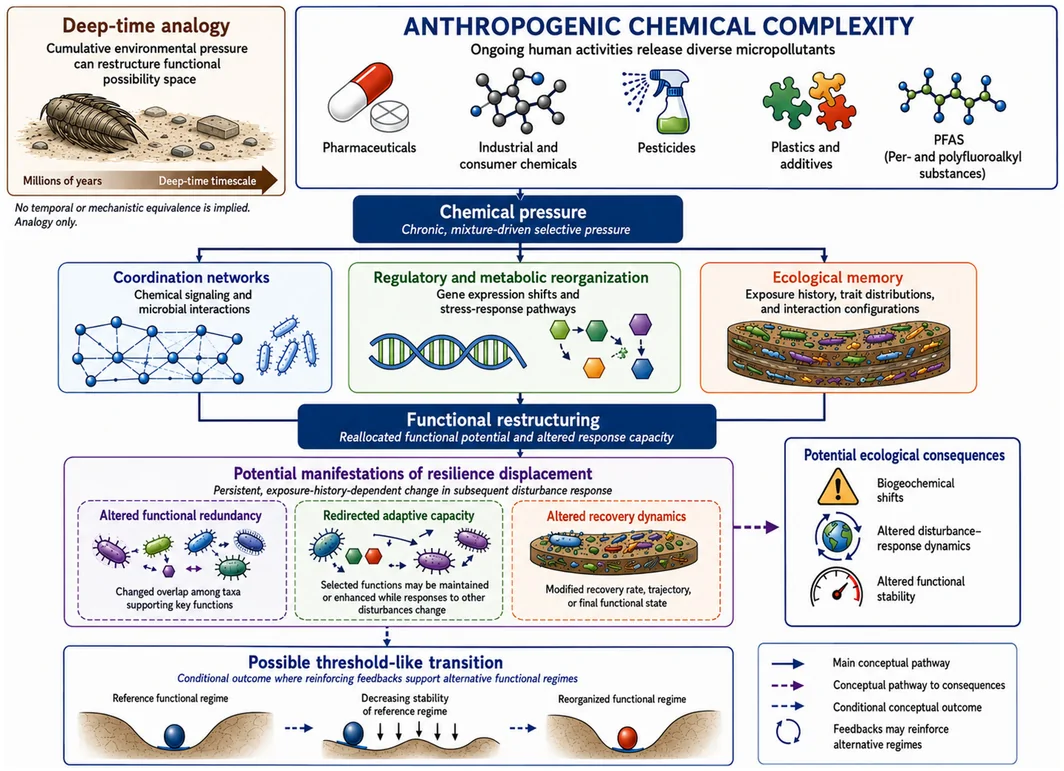
Chemical complexity restructures microbial resilience and may drive threshold transitions. Persistent, low‐dose chemical mixtures can reorganize microbial coordination networks, regulatory and metabolic processes, and ecological memory, thereby altering functional redundancy, adaptive capacity, and disturbance–recovery dynamics. The lower panel illustrates a possible threshold‐like transition where reinforcing feedbacks support alternative functional regimes, rather than a universal response to chronic chemical pressure.

The Anthropocene is characterized not only by climate change, land‐use transformation, and biodiversity loss, but also by an unprecedented expansion of anthropogenic chemical complexity. Microbial communities, which underpin biogeochemical cycling, decomposition, water purification, and many other ecosystem functions, now operate within a continuously renewed background of synthetic compounds that permeate soils, waters, sediments, and engineered systems such as wastewater treatment plants. These chemicals reach microbial communities through interconnected pathways, including micropollutants discharged through wastewater treatment and reuse, agricultural and urban runoff, industrial releases, biosolids application, landfill leachate, and atmospheric transport, creating exposure continua across terrestrial, freshwater, sedimentary, estuarine, and marine environments. Although micropollutants are typically evaluated as individual toxicants, this framing does not capture their ecological reality: persistent, low‐dose mixtures acting as chronic selective environments across ecological and evolutionary timescales. Here we develop a conceptual framework in which anthropogenic chemical mixtures restructure microbial resilience by progressively reorganizing coordination networks, regulatory investment, and ecological memory, thereby shifting the reference states from which microbial communities respond to disturbance before broader ecosystem consequences become detectable. Figure 1 summarizes this framework and the proposed links between chemical complexity, functional restructuring, resilience displacement, and ecosystem‐level consequences.

Deep‐time analogies illustrate how cumulative pressures can restructure ecological possibility space long before collapse becomes visible. Trilobites, which flourished for hundreds of millions of years, did not disappear in a single episode of calamity. Their decline was shaped by long‐term chemical and ecological shifts, including progressive changes in ocean oxygenation, seawater chemistry, and food‐web structure, that slowly narrowed the environmental envelope in which they could function. Recurrent low‐oxygen conditions, increasing ecological competition, and gradual habitat reorganization did not cause immediate collapse, but incrementally reduced their physiological tolerance, ecological flexibility, and capacity for recovery (Feist 1991; McNamara and Feist 2016). Their story reminds us that ecosystems are often reshaped not by sudden forces but by quiet, cumulative pressures that subtly redefine the conditions under which life maintains resilience until persistence becomes impossible. Palaeobiological analyses of trilobites further show that selective extinction can reorganize morphological space itself, altering future evolutionary possibilities without requiring abrupt collapse or proportional losses in taxonomic diversity (Esteve 2025). This history illustrates how persistent, cumulative pressures can progressively reduce resilience and restructure functional possibility space long before overt disruption becomes visible.

The deep‐time analogy is not intended to imply temporal or mechanistic equivalence. Trilobite decline unfolded over geological timescales through interacting climatic, chemical, and ecological pressures, whereas microbial responses to anthropogenic chemicals may emerge over days to decades and across many microbial generations. The intended parallel is narrower: persistent environmental filtering can progressively reorganize functional possibility space and constrain future responses before overt ecological disruption becomes apparent.

Ecological theory distinguishes resistance from two established concepts of resilience. Resistance describes the magnitude of change experienced during a disturbance. “Engineering resilience,” in Holling's terminology, describes the rate at which a system returns toward a specified reference state after disturbance, whereas ecological resilience describes the capacity of a system to absorb disturbance without crossing into a qualitatively different regime maintained by altered structures, processes, or feedbacks (Holling 1973, 1996). In microbial communities, these dimensions may be expressed taxonomically or functionally and need not change in parallel: substantial community turnover may occur while ecosystem functions are maintained through functional redundancy, whereas limited taxonomic change may conceal altered functional performance or recovery capacity (Allison and Martiny 2008). Here, we use microbial resilience to encompass both the recovery of key functions following disturbance and the capacity to remain within a functionally equivalent regime under environmental variability. This interpretation is consistent with broader resilience frameworks that distinguish persistence within a regime from adaptation within that regime and transformation into a qualitatively different system state (Walker et al. 2004). Functional redundancy, response diversity, adaptive capacity, and ecological memory can contribute to these properties by shaping how microbial communities resist, recover from, or reorganize following disturbance.

We define resilience displacement as a persistent, exposure‐history‐dependent shift in the reference state from which a microbial community resists, recovers from, or reorganizes following a subsequent disturbance. It differs from ordinary community or functional turnover under continuous chemical pressure because it cannot be inferred from compositional change alone. Rather, resilience displacement requires evidence that prior chemical exposure alters at least one subsequent response property, such as resistance magnitude, recovery rate or trajectory, restoration of key functions, or the probability of returning to a functionally equivalent regime. The displacement may therefore involve reduced recovery capacity, but it may also reflect redirected resilience, in which adaptation to the prevailing chemical environment enhances selected functions while changing the community's response to other forms of environmental variability.

A primary operational test of resilience displacement would compare chemically conditioned and comparator communities subjected to the same defined disturbance. The clearest candidate metric is the recovery time of a specified microbial function, such as respiration, nutrient transformation, or contaminant degradation, toward a predefined reference range. Evidence of displacement would arise if prior chemical exposure persistently altered the magnitude of functional change during disturbance, the rate or trajectory of recovery, or the final functional state reached. Taxonomic composition, functional‐gene distributions, and interaction‐network properties could provide complementary mechanistic information but should not be interpreted as evidence of resilience displacement without an associated change in disturbance–response dynamics.

Under chronic chemical selection, arising, for example, from sub‐lethal mixtures of micropollutants and other co‐occurring chemical stressors, including antibiotics and metals, microbial trait distributions may be progressively filtered without immediate species loss. In natural ecosystems, this chemical pressure interacts with hydrological variability, nutrient pulses, redox shifts, thermal fluctuations, dispersal, and other ecological processes. Such filtering may alter the functional and regulatory baseline from which microbial communities respond to subsequent disturbance, even while community‐level functions remain apparently stable. Because microbial communities possess substantial metabolic plasticity, chronic exposure to anthropogenic chemical mixtures may expand some capacities, including xenobiotic transformation, detoxification, efflux, stress‐response regulation, biofilm‐associated protection, and resistance‐related functions. Such adaptation may be advantageous when selected traits enhance persistence or allow compounds to be used as resources. Nevertheless, it may reshape community‐level functional allocation. We propose that persistent chemical complexity can produce a shift toward chemically selected traits, with possible collateral effects on broader functional redundancy, response diversity, and temporal flexibility required to buffer natural stochastic disturbances. In this sense, chemical mixtures may not simply narrow microbial functional space; they may redirect it, reorienting the reference state toward a synthetic chemical environment and altering the capacity of microbial systems to respond to stochastic disturbances such as wetting–drying cycles, nutrient pulses, redox shifts, or thermal fluctuations. Rather than treating micropollutants primarily as stressors that alter microbial community composition or function, this perspective reframes persistent anthropogenic chemical mixtures as forces that may reshape the baseline conditions from which microbial resilience emerges. In this view, chemical complexity does more than perturb microbial systems: it can progressively reorganize the functional, regulatory, and ecological‐memory structures through which resilience is built, maintained, and expressed.

Today, as our anthropogenic chemical universe expands (Persson et al. 2022; Richardson et al. 2023), micropollutants constitute a pervasive background of chronic environmental pressure (Balmer et al. 2019; Kalinski et al. 2026; Rayne and Forest 2010; Spindola Vilela et al. 2022). Environmental risk assessment has largely treated these compounds as isolated toxicants, emphasizing acute toxicity and short‐term responses. Such an approach fails to capture the cumulative selective pressures exerted by complex chemical mixtures acting continuously across ecological and evolutionary timescales. Extending resilience theory to anthropogenic chemical complexity reveals how persistent mixtures can restructure microbial stability by altering the conditions under which coordination, adaptation, and recovery occur. In our view, the critical question is not individual toxicity, but how sustained chemical pressure reshapes the reference state from which microbial ecosystems respond to disturbance.

We use “chemical pressure” to denote sustained exposure that modifies microbial processes, microbial interactions, and microbially mediated biogeochemical functions over time. Such pressure increasingly alters biological processes (Baines et al. 2021), ecological interactions (Miller et al. 2018; Parikh et al. 2025) and ecosystem services (Backhaus et al. 2012). Consequently, aquatic systems and soils, which serve as important microbial habitats, are increasingly exposed to anthropogenic chemical pressure (Adyari et al. 2020, 2026; Veloso et al. 2023; Verbücheln et al. 2025). The central argument of this perspective is that micropollutants matter not only because some compounds are acutely toxic, but because persistent low‐dose mixtures can quietly shift the microbial processes that underpin ecosystem resilience.

Micropollutants occur over a spectrum of trace concentrations (Badmus et al. 2021; Kosnik et al. 2022; Scheringer and Schulz 2025; Sigmund et al. 2023), not all of which are deemed environmentally relevant. Their effects cannot be interpreted from individual occurrence and bulk concentrations alone, as biological responses depend on molecular potency, intermolecular interactions, and specific modes of action. Importantly, the pressure experienced by microbial communities is mediated, among many other variables, by matrix‐dependent bioavailability, including the freely dissolved fraction of contaminants and their partitioning into or onto dissolved organic matter, colloids, suspended particles, sediments, sludge flocs, extracellular polymeric substances, and biofilm matrices (Reichenberg and Mayer 2006; Torresi et al. 2017). These compartments can attenuate exposure by reducing the immediately bioavailable fraction, but they can also create localized microenvironments where hydrophobic or particle‐associated chemicals are concentrated and brought into close contact with microbial cells (Naidu et al. 2008). Thus, anthropogenic chemical complexity creates the potential selective landscape, whereas matrix‐mediated bioavailability defines the realized ecological and evolutionary pressure imposed on microbial communities. For example, pharmaceuticals that modulate neurological signaling (Kaushik and Thomas 2019; Romero‐Alfano et al. 2025), surfactants that depolarize microbial cell membranes (Badmus et al. 2021), and persistent PFAS (per‐ and polyfluoroalkyl substances) that give rise to long‐lived transformation products (Evich et al. 2022) illustrate how structurally diverse compounds can exert ecological effects disproportionate to their abundance. Industrial additives, pesticides, polymer by‐products, and human and veterinary pharmaceuticals, among other compounds, form a persistent chemical background layer that is continuously renewed by manufacturing, consumption, and disposal patterns. Crucially, this background layer is not static. Chemical diversity is expanding rapidly: global chemical production has risen nearly fifty‐fold since 1950, and thousands of new substances enter commercial use every year (Persson et al. 2022). Regulatory assessments focusing on single substances overlook the reality that pollutants occur in mixtures, with potentially amplified and complex combined effects. Together with insufficient preventive approaches and an overly optimistic assumption that the environment would absorb and attenuate impacts, this has gradually placed ecosystems under increasing pressure. The expanding chemical diversity in water systems affects microorganisms and microbial communities through mechanisms that extend beyond direct toxicity. Micropollutants can interfere with signaling pathways and disrupt the timing of physiological and ecological processes (Bottalico and Weljie 2021; Zheng et al. 2021), with cascading and potentially emergent effects on microbial community structure, equilibrium, and function that are not yet fully characterized. Such effects are often subtle, occurring at concentrations far below those associated with acute harm, yet they can modify how microorganisms allocate energy, respond to stress, or interact with their environment. Such responses fall outside traditional pollution paradigms and instead reflect small but persistent functional disturbances that accumulate over the ecological and microbial timescales at which communities operate.

## 2 Wastewater Systems as Gateways Between Chemical Use and Environmental Microbiomes

Wastewater systems represent a major, but not exclusive, pathway through which anthropogenic chemicals are collected, transformed, and released into environmental systems (“Time to Get Clean,” *Nature* 2015). Contemporary wastewater treatment plants were designed in an era when the diversity and abundance of anthropogenic chemicals were substantially lower, and many micropollutants are only partially removed by conventional treatment (Christou et al. 2024; Joss et al. 2006; Michael et al. 2013). Biological wastewater treatment systems, mainly based on activated sludge and biofilm processes, host highly adaptive microbial communities with substantial metabolic plasticity. These communities metabolize, transform, or sequester a wide range of chemical compounds. However, such responses are often incomplete and trade‐off‐laden: parent compounds may be converted into transformation products rather than fully mineralized (G. Wu et al. 2025; Yu et al. 2026), while chronic chemical exposure may favor specialized metabolic traits, stress‐response pathways, biofilm‐associated protection, and horizontal gene transfer, potentially at the expense of structural stability or broader functional redundancy.

Engineered wastewater treatment systems are not direct analogues of natural ecosystems. Their hydraulic and solids‐retention times, aeration, biomass management, and disturbance regimes are externally controlled, whereas microbial communities in receiving environments are shaped by hydrological variability, resource pulses, redox fluctuations, dispersal, predation, and other ecological processes. Their relevance to the present framework therefore lies not in serving as universal models of natural microbial resilience, but in providing tractable, intensively monitored settings in which chemical–microbial interactions, adaptive responses, and transformation processes can be examined. Whether comparable mechanisms alter resilience in natural communities must be evaluated independently under the ecological conditions of receiving environments.

As a coupled human–microbial–environmental system, wastewater infrastructure links chemical production, microbial adaptation, and downstream microbiome‐dependent ecosystem processes into a continuous feedback loop. Changes occurring within engineered treatment systems therefore propagate beyond local boundaries, influencing riverine, coastal, and soil microbial processes that underpin biogeochemical cycling (Desiante et al. 2022). In this sense, wastewater systems function not only as treatment facilities, but as arenas where anthropogenic chemical forcing intersects directly with microbial ecology, microbial evolution, and the biogeochemical processes they sustain. Intensifying urbanization, expanding chemical use, and water scarcity–driven wastewater reuse are bringing human populations and microbial ecosystems into progressively closer contact with human‐impacted water flows, amplifying their environmental and public‐health relevance.

Wastewater is a dynamic ecosystem where particles, polymers, molecules, genes, and microbes interact. Wastewater integrates the chemical signatures of households, hospitals, agricultural runoff, and industry into a single complex mixture with which microbes must coexist and to which some can adapt. Two additional dimensions make wastewater a particularly powerful hub of Anthropocene chemical complexity. First, microplastics and other particulate polymers do not act only as passive particles; they provide hydrophobic surfaces that can concentrate micropollutants and support specialized biofilms, creating plastisphere microhabitats in which chemical mixtures, microbial cells, extracellular polymeric substances, and mobile genetic elements are brought into close spatial association (Amaral‐Zettler et al. 2020). Second, wastewater contains sub‐lethal concentrations of antibiotics, biocides, heavy metals, and other co‐selective agents that can favor resistance‐related functions and promote the persistence and horizontal dissemination of antibiotic‐resistance genes and mobile genetic elements (Berendonk et al. 2015). These particulate, chemical, and genomic dimensions mean that wastewater functions not only as a transport route for micropollutants, but also as a site where chemical partitioning, biofilm ecology, co‐selection, and genetic exchange converge.

Within treatment systems, microbes metabolize, transform, or stabilize micropollutants, producing metabolites, hybrid intermediates, or partial breakdown products (Alexander 1981; Kennes‐Veiga et al. 2022). Wastewater treatment microbiota therefore act as the planet's first responders to emerging chemicals and increasingly complex mixtures. This interaction can be viewed as a dynamic interplay between chemical persistence and degradability on the one hand, and microbial community restructuring and genomic evolution on the other. The balance between chemical persistence and microbial transformation determines the potential for pollution attenuation. Understanding these interactions is essential for evaluating both treatment outcomes and downstream ecological impacts. The ecological significance of wastewater systems therefore lies not only in the microbial transformations occurring during treatment, but also in the release of residual compounds, transformation products, particles, and associated microorganisms into receiving environments. Once introduced into rivers, sediments, soils, estuaries, and coastal waters, these inputs encounter microbial communities shaped by distinct hydrological, trophic, and ecological constraints.

## 3 Chemical Pressure Across Natural Microbial Ecosystems

Evidence from natural ecosystems indicates that chronic micropollutant exposure can reorganize microbial communities across multiple environmental compartments. In freshwater systems, chemical pressure has been associated with shifts in prokaryotic community structure across reservoir and riverine gradients, while urbanization can intensify micropollutant effects across planktonic food webs (Adyari et al. 2026). In chemically stressed rivers, responses detected through environmental DNA further indicate that low‐level contaminant mixtures can leave discernible ecological signatures even where acute toxicity is not apparent (Verbücheln et al. 2025). In estuarine sediments, micropollutant‐associated microbial modules have been linked to keystone taxa, suggesting that chemical exposure may alter not only community composition but also the interaction architecture through which community organization is maintained (Veloso et al. 2023).

Comparable patterns are emerging in terrestrial systems. Pesticide residues can alter both taxonomic and functional soil biodiversity (Köninger et al. 2026), while PFAS exposure can reshape soil microbial community structure and metabolic profiles (E. Wu et al. 2023). Together, these studies show that anthropogenic chemical pressure can modify microbial composition, functional potential, and ecological interactions across freshwater, sedimentary, estuarine, and terrestrial environments. Most available evidence, however, documents microbial restructuring rather than directly testing the disturbance–response criteria for resilience displacement defined above.

Evidence from broader chemical‐perturbation studies provides a clearer link between exposure history and subsequent microbial response (Potts et al. 2022). In estuarine and marine sediments, communities with a history of chronic hydrocarbon contamination were more resistant to renewed perturbation, whereas previously unexposed communities underwent stronger compositional restructuring. Despite these contrasting taxonomic responses, phenanthrene degradation was maintained across distinct community configurations, indicating that ecological memory, community reorganization, and functional redundancy can jointly shape microbial responses to chemical disturbance. These findings do not demonstrate resilience displacement directly, but they show that the history and chronicity of chemical exposure can alter the reference state from which natural microbial communities respond to subsequent perturbation (Potts et al. 2022).

## 4 Multiple Dimensions of Micropollutant Influence

Evidence from laboratory, field, and engineered systems demonstrates that micropollutants can alter microbial community composition, signaling pathways, and functional performance. These observations support broader inferences about microbial coordination, resilience, and adaptation. In the following sections, we distinguish between empirically supported effects, strong inferences, and emerging hypotheses regarding long‐term ecological and evolutionary consequences.

One important dimension concerns how micropollutants interfere with microbial communication systems. Microorganisms rely on chemical signals (Amin et al. 2015; Rémy et al. 2018), such as quorum‐sensing molecules, redox cues, and metabolic intermediates, to coordinate behavior and regulate collective functions (Abisado et al. 2018; Liu et al. 2024). Several micropollutants can influence these signaling pathways by affecting the cellular processes that produce, release, or detect these signals (Gagné 2017; Jin et al. 2025; Lami et al. 2023; Yin et al. 2023). These interactions do not produce classical toxic effects, but they can alter microbial coordination and shift community behavior (Kong et al. 2023; Köninger et al. 2026; Lami et al. 2023; Ma et al. 2025; Wang et al. 2024).

Microbial processes are regulated by genetic and physiological mechanisms synchronized with environmental conditions, such as hydrological pulses or nutrient availability, giving rise to temporally structured dynamics. Persistent exposure to micropollutants may impose selective pressures that drive regulatory microbial adaptation and niche specialization within subsets of the microbial community. These pressures can alter cellular regulatory networks, stress‐response pathways, and metabolic allocation patterns, ultimately reshaping community composition and functional succession. Several non‐mutually exclusive mechanisms may underlie this effect. Chronic exposure may divert energy and reducing power from growth, repair, and recovery toward detoxification, efflux, oxidative‐stress management, and damage control (Martinez et al. 2009; Muller et al. 2007; Newsome and Falagán 2021); reset regulatory thresholds so that stress‐response pathways remain partially activated even under otherwise favorable conditions; alter quorum sensing, extracellular polymeric substance production, and biofilm formation, thereby changing community‐level temporal coordination (Kong et al. 2023); and favor taxa whose chemically selected traits improve persistence under contaminant exposure but reduce responsiveness to natural environmental pulses. While mechanistic integration of these processes across temporal scales remains insufficiently resolved, it is hypothesized that their convergence underpins the impact of micropollutants on microorganisms, not only on short timescales, where effects may influence desirable microbially mediated biogeochemical and engineered‐process reactions, but also over the long term, through genomic restructuring and lineage turnover. Such timing‐related effects are not captured by conventional toxicological endpoints, highlighting a critical gap in current assessment frameworks.

Chemical novelty is now an intrinsic feature of microbial life, and it can drive shifts in evolutionary and metabolic trajectories. Established examples include microbial pathways capable of degrading synthetic compounds such as atrazine (de Souza et al. 1996, 1998) and dichlorodiphenyltrichloroethane (DDT) (Bumpus and Aust 1987), while contemporary chemical novelty is increasingly represented by pharmaceuticals and their transformation products (Wu et al. 2025; Yu et al. 2026), PFAS (Evich et al. 2022; E. Wu et al. 2023), polymer additives and plastic‐associated chemicals (Jin et al. 2025; Zhang et al. 2025), and complex multi‐component mixtures. The continual introduction of new pharmaceuticals means that microbial communities are constantly exposed to compounds with no evolutionary precedent. While this chemical innovation is unlikely to slow down, an opportunity lies in designing pharmaceuticals that achieve therapeutic efficacy while minimizing unintended impacts on environmental microbial communities. For several of these compound classes, complete microbial mineralization may be limited or unresolved, but recent metagenomic, multi‐omics, enrichment‐culture, and enzyme‐focused studies show that microbial communities can respond through stress adaptation, altered regulatory pathways, co‐metabolic transformation, and the enrichment of candidate functional genes or taxa (Bumpus and Aust 1987; E. Wu et al. 2023). Comparable processes likely occur continuously in wastewater biofilms, activated sludge, riverine sediments, and soils, where microbes can express alternative catabolic routes, reorganize metabolic interactions, or activate genes that remain silent under stable conditions. Such processes, together with communication, functional synchronization, and genetic diversification, represent ecological dimensions often overlooked in contaminant science. Micropollutants can influence these dynamics by imposing chronic anthropogenic selection pressures, thereby shaping the adaptive repertoire available to microbial communities. Under sustained chemical exposure, microbial resilience is therefore not a static property but an emergent outcome of how functional redundancy and metabolic flexibility are reorganized through selection, determining whether microbial assemblages retain, lose, or redirect biochemical capacities while maintaining ecosystem‐level processes (Scanlon et al. 2025; Stirling 2010).

Micropollutants may also influence ecological memory, understood as the imprint of past conditions stored in microbial gene pools, trait distributions, and established interaction networks (Scanlon et al. 2025; Gajrani et al. 2025). Continuous exposure has the potential to shift the prevalence of specific genes, favor tolerant or stress‐adapted taxa, and stabilize new metabolic and syntrophic configurations. These changes redefine the starting point from which communities respond to future disturbances. In this sense, chemical mixtures can gradually reshape the inherited biological and functional baselines that underpin microbial resilience.

Together, these arguments underlie the vision that micropollutants exert their influence not by producing acute harm but by modifying the conditions under which microbial resilience is built, stored, and expressed.

Where chronic chemical pressure drives microbial systems toward a threshold‐like transition, declining recovery rates and increasing temporal autocorrelation or variance may provide candidate early‐warning signals of reduced resilience, consistent with critical‐transition theory (Scheffer et al. 2009). Such signals are not expected to arise universally, but primarily where reinforcing feedbacks support alternative functional regimes. More broadly, microbial reorganization may be detected through changes in regulatory states, functional‐gene distributions, interaction patterns, and disturbance–recovery dynamics. Communities exposed to sustained chemical mixtures may show altered prevalence of stress‐response, detoxification, efflux, biofilm‐associated, xenobiotic‐transformation, or resistance‐related functions. Because changes in network modularity, connectivity, or functional overlap can have different ecological meanings depending on community composition, exposure history, and environmental context, these properties should be treated as indicators of microbial reorganization rather than as fixed directional predictions. In this framework, mixture‐driven selection is proposed not simply to alter community composition, but to modify the reference state from which microbial communities respond to future disturbance.

A resilience‐oriented monitoring and risk‐assessment framework would integrate chemical‐mixture characterization with longitudinal measurements of microbial structure, function, and disturbance response. It would first define ecosystem‐ and function‐specific reference ranges and characterize exposure in terms of mixture composition, transformation products, and bioavailability. Exposed and comparator communities would then be evaluated through repeated observations and, where feasible, standardized disturbance–recovery assays. Core endpoints would include resistance magnitude, recovery time and trajectory, restoration of key ecosystem functions, persistence of altered functional‐gene distributions, and changes in functional response diversity or interaction‐network organization. This framework would distinguish transient compositional variability from persistent changes in recovery capacity and would complement, rather than replace, conventional toxicity endpoints by testing whether chronic chemical exposure alters the ability of microbial communities to absorb disturbance, restore key functions, or remain within a functionally equivalent regime.

## 5 Why Reframing is Necessary for Science, Policy, and Practice

Current environmental risk assessment treats micropollutants largely as individual substances, tested as single compounds. Yet the ecological effects discussed above arise from interactions among compounds, matrices, microbial traits, and exposure histories. Chemical‐by‐chemical testing and regulation cannot keep pace with the expansion of chemical diversity, nor with the complexity of environmental mixtures. Current environmental risk assessment often misses intermolecular interactions and subtler microbial endpoints, including altered intercellular communication, changes in genetic regulation and plasticity, and longer‐term effects on the functioning of microbial communities and the services they support, because these remain largely invisible to traditional toxicology (Stirling 2010; Van Der Sluijs et al. 2005).

Recognizing micropollutants as subtle ecological drivers with potentially adverse consequences has practical implications, particularly in wastewater systems, where continuously renewed chemical inputs can create highly dynamic evolutionary environments. It reinforces the need for monitoring approaches that holistically characterize the chemical environment, including transformation processes and the emergence of novel, potentially biologically active chemical species.

Failing to account for these cumulative, mixture‐driven effects risks underestimating how chemical pollution may influence ecosystem stability through its microbial foundations.

## 6 Concluding Remarks

Micropollutants are increasingly part of the ecological background against which microbial resilience is now constructed and contested. Reframing them as ecological modulators, rather than isolated toxicants, reveals chemical complexity as more than an environmental burden: it is a force that can progressively reorganize microbial functional redundancy, adaptive capacity, and ecological memory. In the Anthropocene, microbial resilience may therefore be undermined not only by abrupt shocks, but by persistent chemical novelty that quietly alters the conditions under which microbial communities recover, persist, or transform. Integrating these insights will strengthen our ability to anticipate environmental change by detecting microbial restructuring before its consequences become visible at higher levels of ecological organization.

The challenge ahead is therefore not only to detect chemical pollution, but to understand how chemical complexity reorganizes the microbial foundations of ecosystem resilience. This requires moving beyond compound‐by‐compound assessment toward approaches that integrate mixture chemistry, transformation products, bioavailability, microbial network structure, functional genes, horizontal gene transfer, and recovery dynamics under realistic environmental variability. Such a shift is central to Anthropocene governance: chemical design, wastewater treatment, reuse policy, biodiversity protection, and planetary health will increasingly depend on whether we can recognize and manage microbial resilience displacement before its consequences become visible at higher levels of ecological organization.

## Author Contributions

**Celia Manaia:** writing – original draft, writing – review and editing. **Luis Pedro Coelho:** writing – original draft, writing – review and editing. **Despo Fatta‐Kassinos:** conceptualization, investigation, methodology, visualization, writing – review and editing, supervision, writing – original draft. **Andrea Naziri:** writing – original draft, writing – review and editing.

## Conflicts of Interest

The authors declare no conflicts of interest.

## Data Availability

The authors have nothing to report.
